# Unpacking resilience: exploring the link between dissociative responses and psychological resilience in war-affected Palestinians

**DOI:** 10.3389/fpsyt.2025.1678100

**Published:** 2025-10-14

**Authors:** Hamzeh Yacoub, Suheir S. Sabbah, Zaid Yacoub, Rita Yacoub, Khalil Sabbah, Diana Yasin, Yousef B. Ghannam

**Affiliations:** ^1^ Faculty of Medicine, Al-Quds University, Jerusalem, Palestine; ^2^ Psychology Department, Faculty of Education, Al-Quds University, Jerusalem, Palestine; ^3^ Faculty of Medicine, Jordan University of Science and Technology, Irbid, Jordan; ^4^ Department of Mental Health, Faculty of Public Health, Al-Quds University, Jerusalem, Palestine

**Keywords:** peritraumatic dissociation, resilience, war trauma, dissociative symptoms, mental health in armed conflict

## Abstract

**Introduction:**

Peritraumatic dissociation (PD) is a psychological response to trauma involving disruptions in awareness, memory, and identity. While PD is associated with adverse outcomes such as PTSD, the role of resilience in shaping dissociative experiences remains underexplored, particularly in conflict-affected populations.

**Objective:**

To examine the relationship between peritraumatic dissociation and psychological resilience among Palestinians exposed to war-related trauma.

**Methods:**

A cross-sectional survey was conducted among 623 Palestinian participants from Gaza, the West Bank, and Israeli-controlled areas during the December 2024 Gaza-Israel war. Peritraumatic dissociation was assessed using the Peritraumatic Dissociative Experiences Questionnaire (PDEQ), and resilience was measured using the 10-item Connor-Davidson Resilience Scale (CD-RISC-10). Linear and multiple linear regression analyses were used to assess relationships and predictors.

**Results:**

Participants demonstrated moderate levels of both PD (M=28.31, SD=8.44) and resilience (M=32.38, SD=8.92). Regression analysis revealed a significant positive association between resilience and PD (β=0.404, p < 0.001). Females reported significantly higher PD levels than males, while higher education was associated with greater resilience. A history of mental illness predicted both higher dissociation and lower resilience.

**Conclusion:**

Contrary to expectations, resilience was positively correlated with peritraumatic dissociation, suggesting a complex relationship between psychological endurance and acute dissociative responses. Rather than indicating an adaptive role for dissociation, the findings may reflect the intensity of trauma exposure among resilient individuals in conflict zones.

## 1 Introduction

Dissociation is the process by which individuals experience disruption or discontinuity in various aspects of their psychological functioning (1). Dissociation often impacts functions such as memory, perception, awareness, identity, and motor control (2). Trauma survivors may employ dissociation as a defense mechanism, hence increasing the risk of Post-traumatic Stress Disorder (PTSD) (3).

Peritraumatic Dissociation (PD) refers to a range of dissociative experiences that occur during or directly after exposure to a traumatic event, such as war, kidnapping, sexual abuse or violence, rape, forced displacements, and many others (4). Patients with PD may experience emotional numbness, out-of-body experiences, altered perception of time, dissociative amnesia, and disruption in memory and awareness. PD is considered a strong predictor of various trauma-related disorders, including Acute Stress Disorder (ASD), dissociative disorders such as dissociative amnesia and Dissociative Identity Disorder (DID), and is most commonly linked to PTSD (5).

Dissociative symptoms, predominantly PD, have become increasingly important for both the diagnosis and prognosis of PTSD and ASD. A large meta-analysis by Ozer et al. concluded that PD is one of the strongest predictors of PTSD (6). Although PD is not officially recognized as a diagnostic criterion under the Diagnostic and Statistical Manual of Mental Disorders, Fifth Edition (DSM-5) for PTSD, it remains clinically relevant due to strong relationship with DSM-5 symptom clusters for PTSD criteria (7). These include intrusions, avoidance, and cognitive/mood clusters of PTSD symptoms, which are often associated with PD symptoms such as trauma memory disruption, emotional numbing, detachment, and others (8).

Trauma and its psychological aftermath have long been a focus of research, with the development of PD and PTSD being prominent outcomes. However, not everyone exposed to trauma develops PD or PTSD, and individuals may experience varying severity of symptoms, regardless of whether they are exposed to the same traumatic event (9). This discrepancy has led researchers to investigate factors contributing to psychological resilience. While there is no universally agreed definition or measurement, resilience is generally conceptualized as the ability to recover from or adapt successfully to adversity or risk, serving as a protective factor that mitigates the negative psychological consequences of trauma (10).

A recent shift in the mental health field has left a greater focus on positive psychological constructs including resilience, optimism, and happiness which protect individuals from developing mental illness after exposure to stress. These constructs operate through dynamic interactive processes that help individuals return to their previous level of functioning (11, 12). For example, a study by Veronese et al. reported that despite high levels of psychological distress among Gazan university students living under siege, resilience significantly reduced feelings of hopelessness and mental distress (13).

During or immediately after exposure to severe trauma, PD can impair trauma processing ability, as the mind may disconnect from overwhelming or inescapable experiences, thereby increasing the risk of developing PTSD (14). However, many insightful studies suggest that higher levels of resilience may buffer the negative effects of PD and reduce the risk of developing further severe trauma-related disorders such as PTSD (15). Resilience not only allows individuals to return to their pre-trauma state but also enhances adaptive coping mechanisms, social support, emotional regulation, and the capacity to find meaning in the traumatic experiences (16). A recent study found that coping mechanisms during the 2023 Gaza war were largely adaptive, with low levels of catastrophizing, an outcome attributed to high levels of resilience among Gazans (17). Moreover, resilient individuals demonstrate greater neurobiological and psychological flexibility, which may support more effective integration of traumatic memories and reduce the likelihood of dissociative responses (18).

Despite the increasing interest in trauma-related mental health research in Palestine, studies have largely focused on PTSD prevalence and trauma exposure. Very few have examined the relationship between resilience and specific trauma responses such as PD. For instance, Thabet et al. reported high PTSD symptoms rates among children in Gaza, but their study has not assessed dissociative reactions or the mitigating role of resilience (19). Similarly, Khamis et al. reported that adolescents with higher trauma exposure experienced greater psychological distress, but resilience was only examined in relation to anxiety and depression, not dissociation (20). While these two research studies sparked new opportunities to further expand the investigation of trauma-affected individuals, our study focuses on the relationship between PD and resilience during wartime.

Since October 2023, the residents of Gaza and the West Bank have witnessed one of the most violent armed conflicts, which has resulted in more than 50,000 deaths and 90,000 casualties (21). This conflict was accompanied by a severe shortage of food, clean water, medical care, and large-scale destruction of infrastructure and massive displacement. In addition to the direct physical threat, Palestinian civilians endure cumulative and ongoing psychological stressors such as existential uncertainty, loss of loved ones, prolonged grief, and the destruction of homes and communities. On the other hand, cultural factors such as strong family structures, community bonds, and religious beliefs play a central role in shaping Palestinian experience, cope with, and recover from trauma. These factors represent a unique sociocultural element that may influence how both resilience and dissociation manifest in this population, emphasizing the need for culturally informed trauma research in this area of research.

The relationship between PD and resilience remains insufficiently explored, particularly within conflict zones such as Gaza and the West Bank, where chronic warfare conditions prevail. Existing literature has examined this relationship in various trauma contexts, including natural disasters. For example, a study on the 2023 Turkey earthquakes demonstrated that PD was a predictor of higher resilience levels (22). Additionally, resilience has been identified as a protective factor among nurses during the COVID-19 pandemic, mitigating the adverse effects of dissociative coping strategies on secondary traumatic stress (STS) (23, 24). Many studies have also focused on vulnerable populations, such as children and women who have experienced sexual abuse (25).

Regarding armed conflicts, research has been conducted in several countries. For instance, studies on Syrian adolescents (26), Iraq and Afghanistan war veterans (27), and Ukrainian students during the Russian-Ukrainian conflict (28) have explored this relationship. Notably, while some research has addressed the Palestinian context, these investigations have been limited to specific regions and predominantly involved young participants. To date, only one study by Ghannam et al. examined the association between PD and resilience, focusing on adolescents (29).

However, a clear, direct link between PD and resilience has not been firmly established, particularly in populations exposed to chronic political violence and humanitarian crises, such as those in the Occupied Palestinian Territories. While resilience is generally assumed to buffer the effects of trauma and dissociation is typically regarded as maladaptive, some findings suggest that this relationship may be more complex, potentially context-dependent, or even bidirectional (22, 26, 29–31).

To address this gap, this study aims to investigate the relationship between PD and resilience among individuals exposed to war-related trauma among the Palestinian population, while also examining the predictive role of sociodemographic variables. While grounded in the Palestinian context, the findings of this study may have broader implications for understanding trauma response dynamics in populations living under chronic armed conflict and systemic adversity, it also may inform culturally sensitive mental health interventions. In this study, resilience is approached as a dynamic process rather than a fixed trait, involving multiple interacting systems such as social, emotional, and cognitive resources that facilitate recovery and adaptation. Accordingly, this study examined two hypotheses (1): psychological resilience would be significantly associated with PD, though the direction of this relationship was uncertain, and (2) sociodemographic factors (gender, place of residence, and educational level, etc) would significantly predict both PD and psychological resilience.

## 2 Method

### 2.1 Study design

A cross-sectional, survey-based original study was conducted in December 2024 during the Gaza–Israeli war. It examined the association between peritraumatic dissociation and psychological resilience and evaluated sociodemographic predictors among Palestinians exposed to armed conflict.

### 2.2 Participants

Sample size was calculated to be 385 with a 95% confidence interval, and 5% margin of error, assuming a moderate effect size and a large general population. More participants have responded to our survey-based cross-sectional study. A total of 623 Palestinian individuals participated in this cross-sectional study, they were recruited through convenient sampling method, responses were collected through google form surveys distributed to people who met the inclusion criteria: (i) were 18 years of age or older; (ii) identified as Palestinian and resided in the West Bank, Gaza, or Israeli-controlled areas; (iii) had experienced either direct (e.g., physical assault or bombings) or indirect (e.g., witnessing violence related to armed conflict or having close relations -family or friends- who were direct victims) war-related traumas, and (iv) were willing to participate. There were no exclusions based on gender, occupation, or health status, in order to ensure a diverse sample. Informed consent was obtained from all participants before data collection. Those who did not meet the inclusion criteria were excluded. Information on chronic illnesses and history of mental health conditions was collected through self-reported yes/no questions. Trauma exposure was assessed with a single yes/no item (facing a deadly situation in the past month), as well, without further detail on frequency, severity, or type.

### 2.3 Procedure

Data collection took place in December 2024, during the Gaza-Israeli war, via Google Forms questionnaire distributed through social media platforms, and university and NGO email listservs to individuals exposed to direct or indirect war-related trauma. The study procedures received approval from our university Ethics Committee. Informed consent was obtained from all participants, and their privacy was protected in accordance with the ethical guidelines set by the committee.

## 3 Materials

### 3.1 Peritraumatic dissociative experiences questionnaire

Peritraumatic dissociation was measured through the Peritraumatic Dissociative Experiences Questionnaire (PDEQ), conducted by Maramar et al. (32). This is a self-reported questionnaire, consisting of 10 items, has 2 domains the first describing Altered awareness and consists 6 items and the second domain describing Derealization. The items are scored on a 5-point Likert scale ranging from 1 (not at all true) to 5 (extremely true). This scale aims to track the dissociative symptoms that occur through the time of trauma, in which the respondent fills out the questionnaire at the time of the traumatic event.

### 3.2 Connor-Davidson resilience scale

This study used the Arab validated Connor-Davidson Resilience Scale (CD-RISC 10) (33, 34) to measure psychological resilience. It is a validated and reliable 10-item scale, through which respondents rate each item on a 5-point Likert scale from 1 (Strongly disagree) to 5 (Strongly agree). The higher the CD-RISC 10 score, the greater the respondent exhibits resilience. Cronbach’s alpha of CD-RISC 10 scale in this study was 0.93, which reflects an excellent level of internal consistency. Overall, participants demonstrated a moderate level of resilience (M=32.35, *SD=9.00*). As mentioned in **Table 1**.

**Table 1:** Descriptive statistics of the Peritraumatic Dissociative Experiences Questionnaire (PDEQ) and Connor–Davidson Resilience Scale (CD-RISC-10).

Scale	Possible range	Observed range	Mean	*SD*	Coefficient α
PDEQ (Total)	10-50	10-48	28.31	*8.44*	0.90
Domain 1	6-30	6-30	16.53	*5.14*	0.84
Domain 2	4-20	4-20	11.78	*3.91*	0.85
CD-RISC 10	10-50	10-50	32.38	*8.92*	0.93

PDEQ, Peritraumatic Dissociative Experiences Questionnaire. CD-RISC-10=10-item Connor–Davidson Resilience Scale. Domain 1= Altered awareness factor. Domain 2= Derealization factor.

Both scales have no generally established normative data, therefore the scores were classified into three categories: low, moderate, and high categories based on interval scaling principles (35). The total score was categorized into low (10–23), moderate (24–36), and high (37–50) for both scales.

### 3.3 Language and validation of scales

PDEQ and CD-RISC 10 were both distributed in Arabic language. The Arabic version of the CD-RISC scale was validated in a previous study (34). However, to the best of our knowledge, the PDEQ has not been previously validated in Arabic. Therefore, we undertook a full process of validation and adaptation of the scale into Arabic. The original English version was translated into Arabic by a mental health expert who speaks both languages, this was followed by back-translation into Arabic by another bilingual expert, and both were eventually compared to check for conceptual accuracy. Later, two psychology experts reviewed the translated Arabic version for face and content validity, and minor edits were made accordingly to enhance it to fit the Palestinian environment. Consequently, a pilot study was done, results confirmed internal consistency and validity, with Cronbach’s alpha of 0.88, which indicates that the Arabic version is reliable. Eventually, the final results showed strong internal consistency, with a Cronbach’s alpha of 0.90 (**Table 1**).

### 3.4 Data analysis

Data was analyzed via SPSS version 22. There were no missing values. Categorical data were summarized using frequencies and percentages. Advanced analyses were also performed to determine relationships between the variables, including linear regression and Pearson correlation. Statistical significance was determined at p < 0.05, ensuring reliable findings.

## 4 Results

### 4.1 Participant characteristics

The demographic characteristics of the study participants are summarized in **Table 2**. With 623 participants, 36.0% (n=224) were males and 64.0% (n=399) were females. The majority held a bachelor’s degree (67.9%). 57.9% were single and 39.2% were married. According to the place of residence, most participants resided in Gaza (59.2%), followed by the West Bank (35.6%) and (5.1%) in Israeli-controlled areas. In terms of employment, 53.0% were students, 28.5% were employed, and 16.9% were unemployed. A history of mental illness was reported by 10.0%, and 29.9% had faced a deadly situation that thought they might die in the past month.

**Table 2:** Demographic characteristics of the study participants.

Characteristics	Frequency	%
Gender		
Male	224	36.0
Female	399	64.0
Education level		
High school	71	11.3
Diploma	53	8.5
Bachelor’s	423	67.9
Higher education	76	12.2
Marital status		
Single	361	57.9
Married	244	39.2
Divorced	8	1.3
Widow	10	1.6
Place of residence		
Israeli controlled area	32	5.1
West Bank	222	35.6
Gaza	369	59.2
Employment		
Unemployed	105	16.9
Student	330	53.0
Employed	177	28.5
Retired	11	1.8
Monthly salary		
Low	288	46.2
Mid	279	44.8
High	56	9.0
Health condition		
Perfect	79	12.7
Very good	275	44.1
Good	188	30.2
Accepted	66	10.6
Bad	15	2.4
Do you consider yourself religious		
Yes	408	65.5
No	215	34.5
Do you have health insurance		
Yes	393	63.1
No	230	36.9
Do you have a chronic disease		
Yes	94	15.1
No	529	84.9
Do you have a history mental illness in the past		
Yes	62	10.0
No	561	90.0
Did you face a deadly situation in the past?		
Yes	186	29.9
No	437	70.1

### 4.2 The level of peritraumatic dissociation and resilience among the participants

**Table 3** presents the levels of peritraumatic disassociation and resilience among the participants. The mean score for peritraumatic disassociation was 28.31 *(SD=8.44)*, classified into moderate level. 29.1% of the participants reported having a high score, 33.7% having a moderate score, and 37.2% having a low score. Males had a mean score of 27.47 (*SD=7.72*), with 48 (21.4%) reporting high, 89 (39.7%) moderate, and 87 (38.8%) low levels. Females had a slightly higher mean of 28.78 (*SD=8.80*), with 133 (33.3%) high, 121 (30.3%) moderate, and 145 (36.3%) low. By region, Gaza participants had the highest mean PD score at 30.10 (*SD=8.21*), with 128 (34.7%) reporting high, 136 (36.9%) moderate, and 105 (28.5%) low. West Bank participants had a mean of 26.03 (*SD=8.19*), with 50 (22.5%) high, 63 (28.4%) moderate, and 109 (49.1%) low. Those from Israeli-controlled areas had the lowest mean of 23.56 (*SD=7.31*), with only 3 (9.4%) reporting high, 11 (34.4%) moderate, and 18 (56.3%) low PD.

**Table 3:** PD and resilience levels among the participants.

Scale	Mean	*SD*	Participants with high score	Participants with moderate score	Participants with low score
PD	28.31	*8.44*	181 (29.1%)	210 (33.7%)	232 (37.2%)
Male	27.47	*7.72*	48 (21.4%)	89 (39.7%)	87 (38.8%)
Female	28.78	*8.80*	133 (33.3%)	121 (30.3%)	145 (36.3%)
Gaza	30.10	*8.21*	128 (34.7%)	136 (36.9%)	105 (28.5%)
West Bank	26.03	*8.19*	50 (22.5%)	63 (28.4%)	109 (49.1%)
Israeli-controlled areas	23.56	*7.31*	3 (9.4%)	11 (34.4%)	18 (56.3%)
Resilience	32.38	*8.92*	309 (49.6%)	171 (27.4%)	143 (23.0%)
Male	32.21	*9.39*	109 (48.7%)	60 (26.8%)	55 (24.6%)
Female	32.48	*8.65*	200 (50.1%)	111 (27.8%)	88 (22.1%)
Gaza	32.90	*8.78*	189 (51.2%)	107 (29.0%)	73 (19.8%)
West Bank	31.71	*9.02*	106 (47.7%)	58 (26.1%)	58 (26.1%)
Israeli-controlled areas	31.06	*9.56*	14 (43.8%)	6 (18.8%)	12 (37.5%)

PD=Peritraumatic Dissociation (PDEQ). CD-RISC-10=10-item Connor–Davidson Resilience Scale. Classification cutoffs: low (10–23), moderate (24–36), and high (37–50).

**Figure 1:**
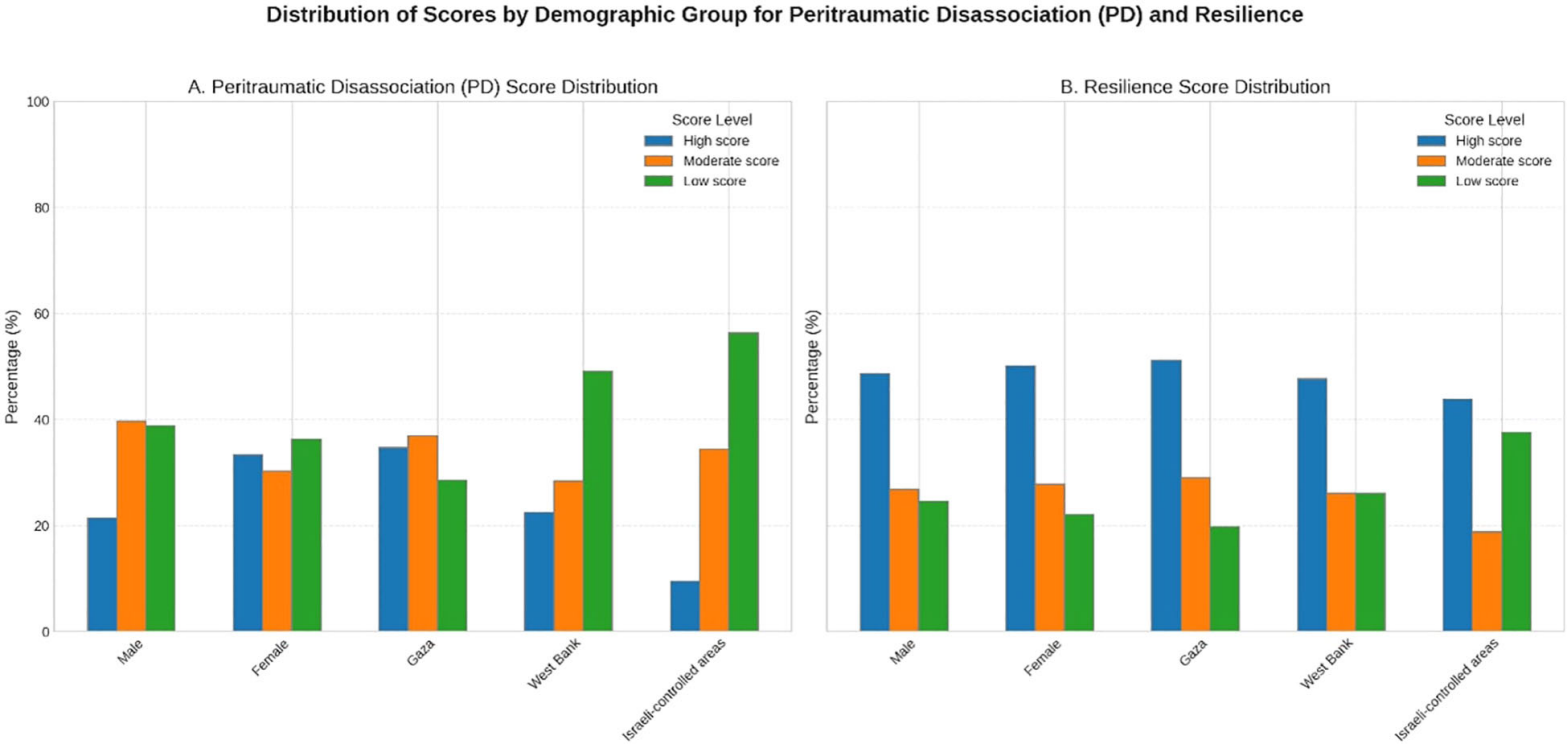
Distribution of peritraumatic dissociation (PDEQ) and resilience (CD-RISC-10) scores by gender and place of residence.

For resilience (CD-RISC 10), the mean score was 32.38 (*SD=8.92*), classified into moderate level. Nearly half (49.6%) of participants reported high resilience, 27.4% moderate resilience, and 23.0% low resilience. Males had a mean score of 32.21 (*SD=9.39*), with 109 (48.7%) high, 60 (26.8%) moderate, and 55 (24.6%) low. Females reported a mean of 32.48 (*SD=8.65*), with 200 (50.1%) high, 111 (27.8%) moderate, and 88 (22.1%) low. Regionally, Gaza participants had the highest mean resilience score at 32.90 (*SD=8.78*), with 189 (51.2%) high, 107 (29.0%) moderate, and 73 (19.8%) low. West Bank participants had a mean of 31.71 (*SD=9.02*), with 106 (47.7%) high, 58 (26.1%) moderate, and 58 (26.1%) low. Those from Israeli-controlled areas had a mean of 31.06 (*SD=9.56*), with 14 (43.8%) high, 6 (18.8%) moderate, and 12 (37.5%) low resilience. As shown in **Figure 1**, the distributions of peritraumatic dissociation and resilience scores varied across gender and regional groups, with Gaza participants showing higher PD levels and individuals with higher education displaying greater resilience.

### 4.3 Regression analysis

Linear and multiple linear regression analysis were conducted (**Tables 4**, **5**). Linear regression showed that resilience was a significant positive predictor of peritraumatic dissociation (β=0.404, p < 0.001), explaining a modest proportion of variance in peritraumatic dissociation (R²=0.163). This indicates that while resilience was a significant predictor, other important factors likely contribute to dissociative responses. The model suggests that, rather than buffering against dissociation, peritraumatic dissociation was linked to increased resilience during trauma exposure, and higher peritraumatic dissociation could be a predictor of higher resilience.

**Table 4:** Linear regression between resilience and peritraumatic dissociation.

Variable	B	β	95% CI for B	p-value	R²	Adjusted R²	F (df)	p (F)
PD	0.43	0.404	0.351-0.503	<0.001	0.163	0.162	121.3 (622)	<0.001**

**P value ≤ 0.05, statistically significant.

**Table 5:** Multiple linear regression analysis.

Variables	Disassociation				Resilience			
	B	SE	β	p-value	B	SE	β	p-value
Age	0.044	0.048	0.053	0.365	0.168	0.049	0.192	0.001**
Gender (Female)	2.404	0.718	0.137	0.001**	-0.355	0.737	-0.019	0.630
Education level								
Bachelor’s	ref							
High School	-1.662	1.132	-0.063	0.143	-5.159	1.162	-0.184	<0.001**
Diploma	-0.995	1.195	-0.033	0.405	-2.318	1.226	-0.073	0.059
Higher education	1.855	1.144	0.072	0.106	1.232	1.174	0.045	0.295
Marital status								
Single	ref							
Married	-1.326	0.937	-0.077	0.158	-2.120	0.961	-0.116	0.028**
Divorced	-6.274	2.928	-0.084	0.033**	-0.719	3.005	-0.009	0.811
Widow	0.619	2.612	0.009	0.813	-1.438	2.681	-0.020	0.592
Place of residence								
Gaza	ref							
West Bank	-4.371	0.825	-0.248	<0.001**	-1.137	0.847	-0.061	0.180
Israeli-controlled areas	-7.093	1.613	-0.186	<0.001**	-2.499	1.656	-0.062	0.132
Employment								
Student	ref							
Unemployed	1.374	1.083	.061	0.205	3.727	1.111	0.157	0.001**
Employed	-2.681	1.034	-0.143	0.010**	-1.324	1.061	-0.067	0.213
Retired	-4.097	2.804	-0.064	0.145	-5.258	2.878	-0.078	0.068
Monthly salary								
Mid	ref							
Low	-0.562	0.727	-0.033	0.440	-0.927	0.747	-0.052	0.215
High	0.262	1.212	0.009	0.829	1.218	1.244	0.039	0.328
Health condition								
Good	ref							
Accepted	-0.260	1.033	-0.009	0.801	-2.130	1.060	-0.074	0.045**
Bad	-3.024	2.143	-0.055	0.159	-3.966	2.199	-0.068	0.072
Do you consider yourself religious (Yes)	-1.595	0.677	-0.090	0.019**	0.334	0.695	0.018	0.631
Do you have health insurance (Yes)	0.102	0.678	0.006	0.880	1.342	0.696	0.073	0.054
Do you have a chronic disease (Yes)	-0.202	1.029	-0.009	0.845	-2.433	1.056	-0.098	0.022**
Did you have a mental illness in the past (Yes)	-4.807	1.233	-0.171	<0.001**	-7.763	1.266	-0.261	<0.001**
Did you face a deadly situation in the past? (Yes)	0.187	0.744	0.010	0.801	0.649	0.763	0.033	0.396

**P value ≤ 0.05, statistically significant.

Multiple linear regression analysis (**Table 5**) was conducted to examine the combined effects of sociodemographic and clinical variables on peritraumatic dissociation and resilience. In these models, all predictors were entered simultaneously, including age, gender, education level, marital status, place of residence, employment status, monthly salary, self-rated health, religiosity, health insurance, chronic disease, history of mental illness, and exposure to a deadly situation. This approach allowed us to assess the independent contribution of each variable while controlling for the others. The results concluded that gender significantly predicts peritraumatic dissociation as Females reported significantly higher dissociation scores than males (B=2.404, p=0.001), but that was not the case for resilience as no significant differences were demonstrated according to gender (p=0.630). According to the Place of residence, participants residing in the West Bank (B=-4.371, p < 0.001) and Israeli-controlled areas (B=-7.093, p < 0.001) reported significantly lower dissociation compared to those residing in Gaza. According to the educational level it was not a significant predictor for peritraumatic dissociation. But for resilience, participants with only a high school education level had lower resilience than those with a bachelor’s degree (B=-5.159, p < 0.001). employment status was not a significant predictor of peritraumatic dissociation. However, Unemployed individuals reported significantly higher resilience (B=3.727, p=0.001) compared to students. A previous history of mental illness was significantly associated with higher dissociation (B=-4.807, p < 0.001) and lower resilience (B=-7.763, p < 0.001). Chronic illness was not significantly related to peritraumatic dissociation, but it is a predictor of lower resilience (B=-2.433, p=0.022). It is also noteworthy that several variables were not significant predictors in the models. Educational level, employment status, chronic illness, monthly salary, and most categories of self-rated health were not associated with peritraumatic dissociation. Similarly, gender, place of residence, and most marital status categories were not significantly associated with resilience.

### 4.4 Correlations between study variables

**Table 6** presents the correlations between the main study variables. A positive correlation appears between resilience and peritraumatic dissociation, revealing a moderate positive correlation (r=0.404, p < 0.001), suggesting that individuals with higher resilience scores tended to report greater peritraumatic dissociation.

**Table 6:** Correlations between peritraumatic dissociation (PDEQ), its domains, and resilience (CD-RISC-10).

Variables	PDEQ	Domain 1	Domain 2	CD-RISC10
PDEQ	1	–	–	–
Domain 1	0.950**	1	–	–
Domain 2	0.911**	0.736**	1	–
CD-RISC10	0.404**	0.390**	0.360**	1

PDEQ=Peritraumatic Dissociative Experiences Questionnaire. CD-RISC-10=10-item Connor–Davidson Resilience Scale. Domain 1= Altered awareness factor. Domain 2= Derealization factor.

** P value ≤ 0.05, statistically significant.

## 5 Discussion

This study investigated the relationship between peritraumatic dissociation (PD) and psychological resilience among Palestinians exposed to direct and indirect war-related trauma. Our results showed that resilience was positively associated with PD, with individuals reporting higher resilience also reporting greater dissociative responses during or immediately after trauma exposure. In addition, several sociodemographic factors shaped trauma responses: females reported higher PD than males, residents of Gaza displayed higher PD compared with the West Bank and Israeli-controlled areas, individuals with higher education reported greater resilience, and participants with a history of mental illness showed both higher PD and lower resilience. These findings highlight that both resilience and dissociation are influenced not only by individual differences but also by broader demographic and contextual factors in populations living under chronic political violence.

### 5.1 PD level as a significant factor in the development of PTSD, depression, and anxiety

According to the literature, two primary trauma responses were identified through which people react to traumatic events; hyperarousal or dissociation (37). The latter can present in different forms as a conversion disorder, presenting as motor or sensory symptoms, dissociative amnesia, dissociative convulsions, or dissociative stupor (38). The dissociative reactions are known associations with increased risk of PTSD, depression, and anxiety (39). As of 2022 after almost one year of the war in Gaza 58 percent of Palestinians in general exhibited symptoms of depression and up to 71 percent in Gaza, these figures are concerning, as prolonged sadness or fear lasting over a month has been strongly linked to an increased risk of developing depression and post-traumatic stress disorder (PTSD) (40, 41). Lack of social support is a known contributor to the development of PTSD (42). In the context of the worsening humanitarian crisis and continuous violence particularly in Gaza, this factor becomes increasingly critical.

Individuals with high anxiety are also more likely to experience severe dissociation and depression, increasing their vulnerability to PTSD (Duncan et al., 2013). This is especially noteworthy in our sample, where participants face daily high levels of stress and anxiety. Notably, PD is also found to be associated with anxiety and stress disorders (45). Overall, PD is closely associated with psychological alterations among people exposed to traumatic events especially in conflict zones.

### 5.2 Association of PD with sociodemographic variables: gender, place of residence, education, and employment status

The ongoing violence continuously exposes civilians to life-threatening danger, loss, and pervasive fear conditions that often exhibit dissociative symptoms. In our study, highest levels of PD were recorded in Gaza (M=30.09, SD=8.21), followed by the West Bank (M=26.03, SD=8.19) and Israeli-controlled areas (M=23.56, SD=7.31). These high scores may be attributed to constant exposure to traumatic events and anticipation of future threats, which have been associated with symptoms of dissociations. It is worth highlighting that the anticipation of trauma is closely associated with increased levels of PD; individuals who experience prolonged anticipation of threat are also associated to exhibit heightened dissociative responses (46), which defines the importance of the study’s findings. Regional variations in PD levels may also be influenced by perceived loss of control, which has been associated to exacerbate dissociative symptoms since it elevates arousal in the face of anticipated trauma (22).

Our findings indicated that females exhibited higher PD levels than males, aligning with previous studies on gender and trauma. Similar results were reported in Turkey among female earthquake survivors (47). These findings are concerning, as peritraumatic and persistent dissociative symptoms are major contributors to the development and persistence of PTSD as discussed before. Our hierarchical regression analysis supported this, with a significant model accounting for 39% of the variance in PTSD symptoms (48). This gender predominance may be attributed to many factors, as stronger perceptions of threat and loss of control are more associated in females, especially in occasions of insufficient social support during wars, as well as gender-specific acute psychobiological reactions to trauma (49).

While gender and place of residence significantly influenced dissociation levels, no significant association was found between PD and educational level, employment status, or chronic illness in our sample. This is an unexpected finding which indicates that these demographic variables may play a lesser role in acute dissociative responses within war-affected populations (46). Nonetheless, this result should be interpreted cautiously as further research may explore potential moderating variables between PD and educational level, employment status, or chronic illness.

### 5.3 Resilience findings

Resilience, assessed using the CD-RISC-10, showed overall moderate levels among participants (M=32.38, SD=8.92), with nearly half (49.6%) demonstrating in the high-resilience range. Participants from Gaza reported slightly higher resilience (M=32.90, SD=8.78) than those from the West Bank (M=31.71, SD=9.02) and Israeli-controlled areas (M=31.06, SD=9.56). This variation may be reflected to the intensity and duration of traumatic events in Gaza, potentially fostering stronger adaptive mechanisms. However, while resilience is protective against PTSD or depression, it does not fully prevent them, confirming prior research (22).

Unlike PD, resilience did not differ significantly by gender; both males and females demonstrated similar coping capacities. However, educational attainment was positively associated with resilience; participants with a bachelor’s degree are reported to have stronger associations with resilience than participants with only a high school education. This result highlight individuals with higher education are strongly associated with resilience as protective effects and coping strategies are found prominent (30, 50).

Interestingly, unemployed participants showed higher resilience than students, possibly due to greater life experience or more developed coping strategies. This finding contrasts with a study in Ukraine, where resilience increased with income. In Gaza, widespread unemployment and poverty may have normalized hardship and encouraged psychological adaptation as well as cultural specificity (51). Although we cannot draw a definitive conclusion whether unemployed participants that show higher levels resilience than students are associated with greater life experience and/or more developed coping strategies. It’s important to note that resilience observed in this study may reflect culturally specific protective factors such as religious faith, normalization of hardships, collective identity, and social lifestyle under apartheid are not fully defined in this study as they are not generalized to other populations. This may give insights for future research studies that includes cross-cultural replications to perceive whether cultural specificity and psychological adaptations play a role in resilience within the Palestinians of Gaza, West Bank, and Israeli-controlled areas.

As expected, a history of mental illness was strongly associated with low levels of resilience, placing this group at higher risk for PTSD, depression, and other mental health disorders (50, 52). Similarly, chronic physical illness was linked to lower resilience, likely due to perceptions of lost autonomy or diminished self-efficacy.

Despite these challenges, the presence of high and moderate resilience in most participants are likely reflected towards personal and community strengths such as religious beliefs, cultural identity, and communal support in sustaining psychological well-being. Although these factors likely play a role, our study was not designed to precisely define their impact, yet these findings align with broader literature emphasizing the protective function of belief systems and social cohesion and its association with resilience (10, 31). Not all conflict-affected populations demonstrate such levels of resilience; for example, a study comparing resilience in Ukraine and Israel found higher resilience in Ukraine, attributed to stronger levels of hope and well-being (51).

### 5.4 Relationship between peritraumatic dissociation and resilience

The findings of our study revealed a significant, unpredicted association between PD and resilience. Linear regression analysis demonstrated that resilience is positively associated with PD (β=0.404, p < 0.001), accounting for 16.3% of the variance in PD. This moderate positive association suggests that individuals with higher resilience scores also possess higher levels of dissociation during trauma exposure. While dissociation is traditionally thought to yield high-risk behaviors in response to trauma and is believed to be associated with lower resilience (53), the pattern detected in this study may reflect a complex, context-dependent dynamic in war-affected populations. We justify this finding as follows: dissociation may act as a defense mechanism against negative emotions creating a psychological buffer and serving as a coping strategy (25, 54, 55), enabling individuals to maintain a level of functional adaptation, in settings such as critical, overwhelming stress, as war trauma, it could translate into enhanced resilience in the longer term, as observed through our findings. Alternatively, more resilient individuals may also possess a deepened awareness of their psychological processes and be more capable of recognizing and reporting the feelings they experience, including dissociative symptoms.

Traditionally, peritraumatic dissociation has been viewed as a maladaptive psychological response that impairs trauma processing and increases the risk of posttraumatic stress symptoms (32, 56). However, the observed positive association between resilience and peritraumatic dissociation challenges this perspective. While resilience is generally seen as an adaptive capacity, its co-occurrence with dissociative symptoms may reflect the intensity and complexity of trauma exposure in war contexts, rather than indicating that dissociation is itself adaptive. Dissociation in such circumstances may be understood as an automatic, non-volitional response to overwhelming threat, which can occur even in otherwise resilient individuals (57). These findings underscore the need for future research studies that focuses on longitudinal data to determine whether dissociation facilitates or buffer long-term adaptations and any associations that may come arise. Additionally, trauma-informed clinical approaches are needed to recognize dissociative symptoms even in individuals with strong coping capacities.

### 5.5 Implications for intervention

The study’s findings highlight the need for context-driven interventions that detect dissociation as both an adaptive survival mechanism and a potential result in war-affected populations. Given the paradoxical link between resilience and peritraumatic dissociation, interventions should validate and consider dissociation as a short-term coping strategy while gradually integrating trauma-informed practices to foster long-term emotional regulation. As women are generally more susceptible to psychological disturbances, and to dissociation as observed in our study, we recommend that gender-sensitive approaches be established in such affected populations, including trauma-focused therapy, to address women’s elevated dissociation levels and gendered trauma experiences. Geographically, prioritizing community-based programs in high-exposure regions to alleviate structural inequities can enhance collective resilience. Campaigns focused on enhancing social ties and building resilience should also take place. Ultimately, interventions must adjust adaptive coping with sustainable resilience-building, anchored in cultural relevance and collaborative, cross-sectoral partnerships.

### 5.6 Strengths and limitations

This study has several notable strengths, including its large, diverse sample (*N*=623) representing key demographics across conflict-affected regions (Gaza, West Bank, Israeli-controlled areas), which enhances ecological validity. It used validated, reliable measures (Cronbach’s α ≥ 0.84) for both peritraumatic dissociation and resilience, which strengthens internal consistency. Additionally, socio-demographic, geographic, and health-related variables provide a broader view of dissociation and resilience determinants. The integration of linear and multiple linear regression models advances understanding of predictors, such as gender, residence, and mental health history, offering actionable insights for targeted interventions.

However, limitations must be acknowledged. The cross-sectional design precludes causal inferences, and the observed association between resilience and peritraumatic dissociation should be interpreted as correlational only. Second, Although Palestine has a youthful demographic structure, recruitment relied on convenience sampling method, which likely introduced selection bias and led to an overrepresentation of younger, highly educated participants and students, this imbalance nonetheless limits generalizability to the wider population. Third, trauma type, frequency, severity, and duration were not collected. Which restricts our ability to study different types of trauma exposure. Additionally, although the Peritraumatic Dissociative Experiences Questionnaire (PDEQ) is designed for use at the time of a discrete traumatic event, our data collection occurred during an ongoing war. Consequently, some responses may have reflected both acute and cumulative dissociative experiences, potentially confounding interpretation of results. And our study did not include a clinical screening or diagnostic assessment for Post-Traumatic Stress Disorder (PTSD), which limits our ability to contextualize peritraumatic dissociation in relation to PTSD development. Future research in conflict settings should aim to incorporate longitudinal designs, more detailed trauma exposure measures, and clinician-administered screening to strengthen interpretive depth and clarify the relationship between peritraumatic dissociation and resilience.

## 6 Conclusion

This study explored the complex relationship between peritraumatic dissociation and psychological resilience in a war-exposed Palestinian population. Findings revealed a moderate positive correlation between PD and resilience, suggesting that dissociative responses may, under extreme stress conditions such as war, serve a paradoxical role in supporting psychological adaptation. Sociodemographic factors, such as gender, place of residence, educational level, and mental health history, significantly influenced dissociation and resilience scores. These findings challenge conventional views that dissociation is inherently maladaptive, highlighting its potential short-term protective function within chronic conflict settings. The study underscores the importance of culturally informed mental health interventions that recognize both the risks and adaptive aspects of dissociation while promoting long-term resilience and recovery.

## Data Availability

The raw data supporting the conclusions of this article will be made available by the authors, without undue reservation.
